# The presence of circulating total DNA and methylated genes is associated with circulating tumour cells in blood from breast cancer patients

**DOI:** 10.1038/sj.bjc.6605013

**Published:** 2009-04-14

**Authors:** I Van der Auwera, H J Elst, S J Van Laere, H Maes, P Huget, P van Dam, E A Van Marck, P B Vermeulen, L Y Dirix

**Affiliations:** 1 Translational Cancer Research Group, Laboratory of Pathology, University of Antwerp/University Hospital Antwerp; Oncology Centre, General Hospital St-Augustinus, B2610 Wilrijk, Belgium

**Keywords:** methylation, circulating tumour cells, circulating DNA, breast cancer

## Abstract

Circulating tumour cells (CTC) and tumour-related methylated DNA in blood have been separately assessed for their utility as a marker for subclinical metastasis in breast cancer. However, no studies have looked into the relation between the both molecular markers in this type of cancer. In this study, we investigated the correlations between total/methylated DNA and CTC in the blood from metastatic breast cancer patients. We simultaneously obtained whole blood, plasma and serum samples from 80 patients and 20 controls. The CellSearch System was used to enumerate CTC in blood samples. Plasma total DNA levels were determined by a QPCR method. Sera were analysed by methylation-specific QPCR for three markers: *adenomatous polyposis coli* (*APC*), *ras association domain family protein 1A* (*RASSF1A*) and *oestrogen receptor 1* (*ESR1*). Total DNA levels in patients were significantly increased when compared with controls (*P*<0.001) and correlated with the number of CTC (*r*=0.418, *P*<0.001). Hypermethylation of one or more genes was detected in 42 (53%) serum samples from breast cancer patients and in three (16%) serum samples from controls (*P*=0.003). *APC* was hypermethylated in 29%, *RASSF1A* in 35% and *ESR1* in 20% of breast cancer cases. Detection of a methylated gene in serum was associated with the detection of CTC in blood (*P*=0.03). The detection of large amounts of circulating total/methylated DNA correlated with the presence of CTC in the blood from patients with breast cancer. This can be interpreted in two ways: (a) CTC are a potential source of circulating tumour-specific DNA; (b) high numbers of CTC and circulating methylated DNA are both a phenotypic feature of more aggressive tumour biology.

Distant metastasis is the leading cause of cancer-related death in breast cancer, but early spread of tumour cells usually remains undetected even by high-resolution imaging technologies. With the use of traditional prognostic factors, it is still not possible to reliably identify those breast cancer patients who will eventually relapse with metastatic disease and are in need of adjuvant therapy. Therefore, the development of new molecular staging methods enabling individual risk assessment is of utmost importance. As haematogenous dissemination of tumour cells is the main mechanism for distant metastasis, the assessment of cancer patients' blood may be a highly desirable approach for detecting systemic tumour cell spreading (Pantel *et al*, 1999).

Several studies have suggested that the presence of circulating tumour cells (CTC) in patients' bone marrow or blood represents a promising marker for current risk classification systems for breast cancer. Recently, we showed that the presence of CK19+ disseminated tumour cells in the bone marrow by reverse transcriptase–PCR is an independent prognostic factor in untreated patients with breast cancer (Benoy *et al*, 2006). Moreover, a pooled analysis involving 4703 patients indicated that the presence of micrometastasis in the bone marrow at the time of diagnosis of breast cancer is an independent predictor of poor prognosis (Braun *et al*, 2005). CTC detection by the CellSearch System (Veridex LLC, Warren, NJ, USA), developed to automatically enrich and immunocytochemically detect CTC from peripheral blood, has been shown to provide significant prognostic information for patients with metastatic breast cancer starting first-line therapy (Cristofanilli *et al*, 2004, 2005). In latter studies, the persistence of CTC at 3 to 4 weeks after the treatment was started and at the time of restaging continued to be significantly associated with prognosis, particularly in women with hormone receptor-negative disease and women who were receiving chemotherapy.

Epigenetic changes, such as DNA methylation, are one of the most common molecular alterations in human neoplasia (Egger *et al*, 2004), including breast cancer (Widschwendter and Jones, 2002). DNA methylation refers to the addition of a methyl group to the cytosine ring of those cytosines that precede a guanosine (referred to as CpG dinucleotides) to form 5-methylcytosine. CpG dinucleotides are found at increased frequency in the promoter region of many genes, and methylation in the promoter regions is frequently associated with gene silencing (Leonhardt and Cardoso, 2000). Several studies have shown that tumour-specific epigenetic alterations can be detected in DNA recovered from plasma or serum of patients with various malignancies (Wong *et al*, 1999; Silva *et al*, 1999a; Sanchez-Cespedes *et al*, 2000), a finding that may be of particular clinical interest in view of molecular diagnosis and prognosis. Increased concentrations of free DNA are detected in the blood of many cancer patients, whereas only small amounts of free circulating DNA are found in healthy individuals (Leon *et al*, 1977). The serum of breast cancer patients contains, on an average, approximately four times more free DNA compared with that of healthy individuals (Gal *et al*, 2004). Recently, investigators have shown that hypermethylation of *ras association domain family protein 1A* (*RASSF1A*) and/or *adenomatous polyposis coli* (*APC*) identified in serum DNA from breast cancer patients is associated with a worse outcome (Müller *et al*, 2003). Furthermore, methylated *RASSF1A* and *neurogenic differentiation 1* gene promoters in serum are candidate biomarkers for monitoring the efficacy of adjuvant therapy in breast cancer patients (Fiegl *et al*, 2005, 2008).

The precise mechanism by which DNA is released into the bloodstream still remains enigmatic. The most common hypothesis advanced for circulating DNA in the blood of cancer patients is that it is because of the lysis of CTC (Stroun *et al*, 2000). Other possible sources include DNA leakage from cells as the result of tumour necrosis or apoptosis, or spontaneous release of DNA into the circulation from primary and metastatic tumours (Stroun *et al*, 2000). Although many studies have suggested the usefulness of CTC or cell-free DNA as a surrogate marker of subclinical metastasis for breast cancer (Taback *et al*, 2001; Huang *et al*, 2006; Ntoulia *et al*, 2006; Wong *et al*, 2006; Wülfing *et al*, 2006; Xenidis *et al*, 2006; Quintela-Fandino *et al*, 2006), few studies have looked into the relation between CTC and cell-free DNA in this type of cancer (Schwarzenbach *et al*, 2004). A combined molecular assessment of the circulating DNA and CTC could improve the evaluation of cancer stage and overall prognosis in breast cancer.

The aim of this study was to identify tumour-specific epigenetic alterations in the cell-free DNA found in the peripheral blood of breast cancer patients and to assess whether a correlation exists between total or tumour-specific methylated DNA and the detection of CTC in peripheral blood of patients with metastatic breast cancer. We decided to investigate these molecular markers in women with metastatic disease because CTC and the circulating DNA levels are known to be higher in these patients.

## Materials and methods

### Patients and sample collection

We prospectively obtained matched peripheral blood, serum and plasma samples from 80 patients with breast cancer and 20 healthy volunteers. All patients gave informed consent for the use of their blood specimen and the examination of blood samples was carried out after approval from the Institutional Review Board of the General Hospital Sint-Augustinus (Wilrijk, Belgium). The patient population consisted of four patients with localised breast cancer (group A), 60 patients with metastatic breast cancer receiving treatment (group B) and 16 patients presenting in our clinic with untreated metastatic breast cancer (group C). Clinicopathological variables are listed in Table 1. The median age of the control population was 39 (range, 25–54) years and 62 (range, 34–85) years in the breast cancer population.

Blood samples from patients with metastatic disease were taken during the course of treatment. Disease status was assessed using the RECIST (Response Evaluation Criteria in Solid Tumours Group) criteria without the knowledge of the patients' CTC or circulating DNA results (Therasse *et al*, 2000). Stable disease was measured up to 8 weeks after the initiation of therapy.

In 18 cases, the corresponding breast cancer tissue samples were available for analysis. All tumour tissues were obtained immediately after surgical resection and snap frozen in liquid nitrogen.

### Quantitative analysis of total DNA

Blood (4.5 ml) from each donor was collected in 0.129 M sodium citrate-containing tubes (Becton Dickinson, Franklin Lakes, NJ, USA) and centrifuged (2000**g**, 10 min) at room temperature. Then, plasma was aliquotted and cryopreserved at −80°C until use. DNA was extracted from 200 *μ*l of plasma using the JETQUICK Blood and Cell Culture Kit (Genomed, Löhne, Germany) according to the manufacturer's protocol, with an elution volume of 100 *μ*l. Plasma DNA concentration was measured by a real-time quantitative PCR assay for the human telomerase reverse transcriptase gene using the Quantifiler Human DNA Quantification Kit (Applied Biosystems, Foster City, CA, USA). The assay included two primers and a FAM-labelled fluorescent TaqMan probe for the target gene, and two primers and a VIC-labelled fluorescent TaqMan probe for an internal control DNA sequence. For construction of the calibration curve, we generated a standard curve using three-fold serial dilutions of known concentrations of DNA (50, 16.67, 5.56, 1.85, 0.62, 0.21 and 0.023 ng *μ*l^–1^). PCR was carried out in a final reaction volume of 25 *μ*l and contained 12.5 *μ*l of Quantifiler PCR Reaction Mix (Applied Biosystems), 10.5 *μ*l of Quantifiler Human Primer Mix (Applied Biosystems) and 2 *μ*l of extracted DNA. Each sample was analysed in duplicate.

The mean quantity of each duplicate calculated by the 7900 sequence detection system software was used for further analysis. The concentration, expressed in nanograms per millilitre, was calculated using the following equation: C=Q × V_DNA_/V_PCR_ × 1/V_ext_ × 1000, where C=target concentration in plasma (nanograms per millilitre); *Q*=target quantity (nanograms); V_DNA_=total volume of extraction (100 *μ*l); *V*_PCR_=volume of DNA solution used per PCR reaction (2 *μ*l); and *V*_ext_=volume of plasma extracted (200 *μ*l).

### Methylated DNA assay

Blood (8 ml) from each donor was collected in serum separator tubes (Becton Dickinson) and centrifuged (2000**g**, 10 min) at room temperature. Then, serum was aliquotted and cryopreserved at −80°C until use. Genomic DNA was extracted from 1 ml of serum using the ZR Serum DNA Kit (Zymo Research, Orange, CA, USA), with an elution volume of 35 *μ*l. Genomic DNA from frozen breast cancer tissues was isolated using the QIAamp DNA Mini Kit (Qiagen, Valencia, CA, USA). Sodium bisulphite conversion of extracted DNA was conducted using the EZ DNA Methylation Kit (Zymo Research). Sodium bisulphite-converted DNA was analysed by real-time qMSP. Two sets of primers and probes, designed specifically for sodium bisulphite-converted DNA, were used: a methylated set for the genes of interest and a reference set, *β-actin* (*ACTB)*, to normalise for input DNA. Fluorogenic probes and PCR primer sets for *APC* (Usadel *et al*, 2002), *RASSF1A* (Lehmann *et al*, 2002), *oestrogen receptor 1* (*ESR1*) (Eads *et al*, 2001) and *ACTB* (Eads *et al*, 2000) were custom synthesised by Applied Biosystems. Fluorogenic PCRs were carried out in a reaction volume of 25 *μ*l in 96-well plates in a 7900HT Sequence Detector (Applied Biosystems). PCR was carried out in separate wells for each primer/probe set, and each sample was run in duplicate. The final reaction mixture consisted of 600 nmol l^–1^ of each primer, 200 nmol l^–1^ of probe and 12.5 *μ*l of Universal Master Mix (Applied Biosystems). Five *μ*l of the treated DNA solution was used in each real-time MSP reaction. Thermal cycling was initiated with a first denaturation step of 95°C for 10 min. The thermal profile for the PCR was 95°C for 15 s and 60°C for 1 min. Data obtained during 50 cycles of amplification were analysed. Each plate included water blanks, a positive control and a negative control. DNA isolated from normal peripheral lymphocytes from healthy individuals served as a negative methylation control. *In vitro* methylated human DNA (Zymo Research) was used as the positive methylation control.

The ratio between the values obtained in the two TaqMan analyses was used as a measure for the degree of methylation of the target gene. The percentage of fully methylated molecules at a gene locus was calculated by dividing the *gene* : *ACTB* ratio of a sample by the *gene* : *ACTB* ratio of fully methylated human DNA and multiplying by 100. We use the abbreviation percentage of methylated reference (PMR) to indicate this measurement. A gene was deemed methylated if the PMR value was >0.

### CTC assay

Peripheral blood (10 ml) was collected from each donor into CellSave blood collection tubes (Immunicon Inc., Huntingdon Valley, PA, USA), which are evacuated blood-draw tubes containing EDTA and a cellular preservative, and processed within a maximum of 72 h after blood drawing (at room temperature).

Circulating tumour cells were enumerated with the CellSearch System (Veridex, Warren, NJ, USA) as described by Allard *et al* (2004). Briefly, 7.5 ml of blood were gently mixed with 6.5 ml of dilution buffer, centrifuged (800 × **g**, 10 min, gentle deceleration) at room temperature and transferred into the CellTracks AutoPrep system (Veridex, Warren, NJ, USA). After aspiration of the plasma and dilution buffer layer, anti-EpCAM antibody-coated ferrofluids were added. After incubation and magnetic separation, unbound cells and remaining plasma were removed, and ferrofluid-labelled cells were re-suspended in buffer, permeabilised, and fluorescently labelled using phycoerythrin-conjugated anti-cytokeratin antibodies recognising cytokeratins (predominantly cytokeratins 8, 18 and 19) to specifically identify epithelial cells, an antibody against CD45 conjugated with allophycocyanin to identify WBC and a nuclear dye (4′,6-diamidino-2-phenylindole (DAPI)) to fluorescently label the cell nuclei. The sample was transferred automatically to a cartridge in a MagNest (Veridex, Warren, NJ, USA), where the immunomagnetically labelled cells move to the surface caused by the strong magnetic field of the MagNest device. The MagNest was placed on the CellTracks Analyzer II (Veridex, Warren, NJ, USA), a four-color semi-automated fluorescence microscope, and image frames covering the entire surface of the cartridge for each of the four fluorescent filter cubes were captured. The captured images containing objects that met predetermined criteria were automatically presented in a web-enabled browser from which final selection of cells was made by the operator. The main criteria for an object to be defined as a CTC included round to oval morphology, a visible nucleus (DAPI positive), positive staining for cytokeratin and negative staining for CD45. Results of cell enumeration were expressed as the number of cells per 7.5 ml of blood, and a cutoff of ⩾2 CTC was chosen to define the test as positive. Each sample was analysed independently by two readers (HJ Elst and PB Vermeulen). Questionable interpretations were evaluated again until consensus was reached.

### Statistical analysis

We used Pearson's *χ*^2^ or, in the case of low frequencies per cell, Fisher's exact method to test associations between categorical variables. The Mann–Whitney *U*-test or the Kruskal–Wallis test was used to assess the differences between non-parametric distributed variables. A two-sided *P*⩽0.05 was considered to be statistically significant. All statistical calculations were carried out using SPSS, version 11.0 (SPSS Inc, Chicago, IL, USA).

## Results

### Total DNA concentrations in plasma from healthy volunteers and breast cancer patients

**Figure 1:**
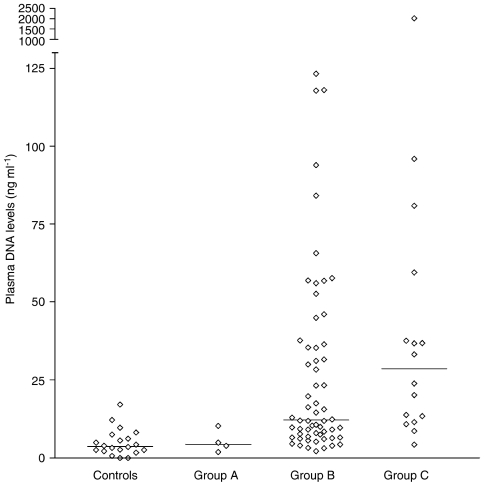
Concentration of circulating DNA in the plasma of different patient groups and controls. Median DNA concentrations are indicated on the graph.

The median concentration of plasma DNA in the healthy controls (*n*=20) was 4 ng ml^–1^ (range 0–17 ng ml^–1^). The median value for breast cancer patients (*n*=80) was 13 ng ml^–1^ (range 2–2027 ng ml^–1^). The median values and ranges for the different groups of breast cancer patients were as follows: 7 (4–12) ng ml^–1^ for group A (*n*=4), 12 (2–123) ng ml^–1^ for group B (*n*=60) and 29 (4–2027) ng ml^–1^ for group C (*n*=16) (Figure 1). The difference between the patients and the controls was statistically significant (*P*<0.001). Differences between plasma DNA concentrations in different patients groups also reached statistical significance (*P*=0.04). The largest differences were measurable between groups A and C (*P*=0.02) and groups B and C (*P*=0.06).

The plasma DNA levels were significantly correlated with CA15.3 levels (*r*=0.276, *P*=0.02) and tended to be correlated with tumour progression, although this was not statistically significant: median total DNA levels were 11 (range, 3–94) ng ml^–1^ in patients with stable disease (*n*=27) and 20 (range, 2–2027) ng ml^–1^ in patients with progressive disease (*n*=46) (*P*=0.09). Plasma DNA concentrations were positively associated with patient age (*r*=0.298, *P*=0.007). No associations with ER, PR, HER2 and p53 were found.

**Figure 2:**
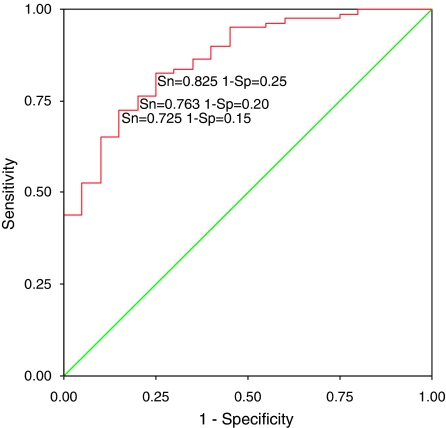
Receiver-operating characteristics curve for plasma total DNA concentration in 20 healthy individuals and 80 patients with breast cancer, plotting the true positive rate (sensitivity) against the false positive rate (1-specificity) for the different possible cut-off points. The area under the curve was 0.865, suggesting a good discriminative power of the molecular assay.

Receiver-operating characteristics (ROC) curve analysis was carried out to define cutoff DNA levels for the prediction of malignancy (Figure 2). The area under the ROC curve assessing plasma DNA concentration was 0.865 (95% confidence interval, 0.782–0.948), suggesting a good discriminative power of the molecular assay. Table 2 lists several cut-off points of DNA values used for the generation of the ROC curve with their sensitivity and specificity. The highest accuracy was obtained at the cut-off point of 8.275 ng ml^–1^ with a sensitivity of 72.5% and a specificity of 85%. We divided patients into high plasma DNA level group (HDNA) and low plasma DNA level group (LDNA) according to the cut-off value identified in the ROC curve analysis. Overall, 85% of patients with progressive disease were in the HDNA group, whereas there were only 59% with stable disease in the HDNA group (*P*=0.01) (Table 3a). Again, no associations were found with ER, PR, HER2 and p53.

### Detection of tumour-related methylated DNA in serum

**Figure 3:**
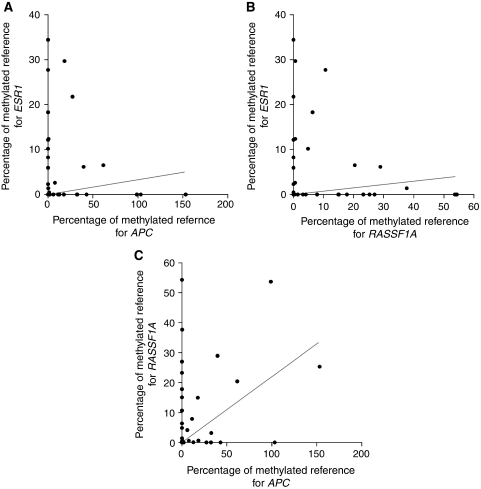
Correlations between methylation levels for the three genes. A significant correlation was observed between percentage of methylated reference (PMR) values for *adenomatous polyposis coli* (*APC*) and *ras association domain family protein 1A* (*RASSF1A*) (**C**) and between PMR values for *RASSF1A* and *oestrogen receptor 1* (*ESR1*) (**B**). No clear correlation was observed between PMR values for *APC* and *ESR1* (**A**).

**Figure 4:**
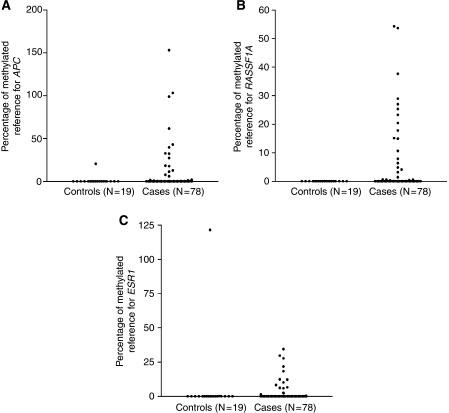
Methylation levels of (**A**) *adenomatous polyposis coli* (*APC*), (**B**) *ras association domain family protein 1A* (*RASSF1A*) and (**C**) *oestrogen receptor 1* (*ESR1*) in serum DNA of healthy controls (*n*=19) and breast cancer patients (*n*=79). Differences in percentage of methylated reference (PMR) values between patients and controls were examined with the Mann–Whitney *U*-test. For *APC* and *RASSF1A* significantly higher PMR values were observed in patients versus controls (*P*=0.04 and *P*=0.003, respectively). For *ESR1*, differences in PMR values between patients and controls did not reach statistical significance (*P*=0.33).

We selected a panel of three genes, namely, *APC*, *RASSF1A* and *ESR1*, for the detection of tumour-specific methylated DNA in serum samples from healthy controls (*n*=19) and breast cancer patients (*n*=79). Median PMR values of *APC* and *RASSF1A*, but not of *ESR1*, were significantly higher among breast cancer cases as compared with controls (*P*<0.05). Methylation of *APC* was positively associated with the methylation of *RASSF1A* (*r*=0.372, *P*=0.001), but not with methylation of *ESR1* (Figure 3). The distribution of PMR in the patient and control groups is shown for all three genes in Figure 4. PMR values of *APC* (*r*=0.398, *P*=0.001), *RASSF1A* (*r*=0.551, *P*<0.001), but not of *ESR1* (*r*=0.078, *P*=0.52), were positively correlated with levels of the tumour marker CA15.3. No associations were found between PMR values of the three genes and age of the patient.

Detection of any level of aberrant methylation (PMR>0) resulted in a specificity for detecting breast cancer of 95% for *APC*, 100% for *RASSF1A* and 89.5% for *ESR1*. The frequency of methylation in serum DNA of *APC* and *RASSF1A* was significantly higher among women with breast cancer as compared with controls: *APC*, 29 versus 5% (*P*=0.03) and *RASSF1A*, 35 versus 0% (*P*=0.002). However, the association of methylation in serum DNA of *ESR1* with case status did not reach statistical significance: *ESR1* was hypermethylated in 20% of breast cancer patients and 10.5% of healthy controls (*P*=0.33). Of the 79 patients with breast cancer, 7 (9%) had all the three methylated markers, 18 (23%) had at least two methylated markers and 42 (53%) had at least one methylated marker in serum. Of all two-marker combinations, the combination of *APC* and/or *RASSF1A* methylation was most frequently observed, in 47% of breast cancer cases. Methylation of multiple genes in serum DNA was not detected in any of the 19 controls. The difference in the number of methylated genes between breast cancer patients and controls was statistically significant (*P*=0.003).

The detection of at least one methylated marker in serum was associated with the disease status (*P*=0.02) (Table 3b). Hypermethylation of both *APC* and *RASSF1A*, but not of *ESR1*, was more frequently detected in patients with progressive disease as compared with patients with stable disease (*P*=0.08 and *P*=0.001, respectively). Hypermethylation of *RASSF1A* was significantly associated with ER expression (*P*=0.01) and HR status (*P*=0.007), which was defined as positive if ER and/or PR were positive (Table 2). No associations were found with PR status, HER2 status, triple negative (ER–, PR– and HER2–) tumours and p53 status. No significant associations were found between hypermethylation of *ESR1* and *APC* and any of the clinicopathological variables (Table 1). Although there were no statistically significant differences in detection of aberrant methylation by age among breast cancer patients, each gene was more likely to be aberrantly methylated in older (>60y) compared with younger participants with breast cancer (Table 4).

**Figure 5:**
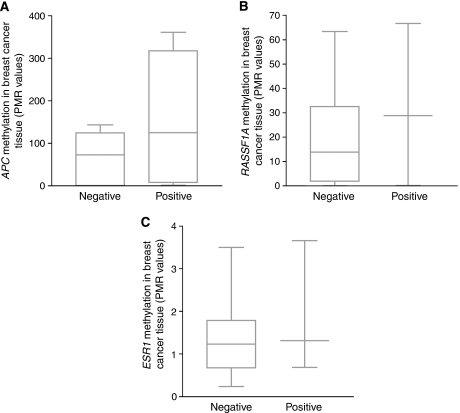
Comparison of percentage of methylated reference (PMR) values in breast cancer tissue between unmethylated (negative) and methylated (positive) serum samples for (**A**) *adenomatous polyposis coli* (*APC*), (**B**) *ras association domain family protein 1A* (*RASSF1A*) and (**C**) *oestrogen receptor 1* (*ESR1*). Differences in PMR values between unmethylated and methylated serum samples were examined with the Mann–Whitney *U*-test. For none of the genes did these differences reach statistical significance.

In 18 cases, the corresponding breast cancer tissue samples were available for analysis. *ESR1* was methylated in all tissue samples, whereas *APC* was unmethylated in one case and *RASSF1A* in three cases. All of the genes found to be methylated in serum samples were also methylated in the corresponding tissue sample. Comparison of PMR values of breast cancer tissue samples between unmethylated and methylated serum samples for each investigated gene showed no significant results (Figure 5). Median PMR values in, respectively, unmethylated and methylated serum samples were 72 (range, 0–143) and 125 (range, 1–361) for *APC* (*P*=0.21), 12 (range, 0–63) and 48 (range, 1–67) for *RASSF1A* (*P*=0.127) and 1.2 (range, 0.2–3.5) and 1.3 (range, 0.7–3.7) for *ESR1* (*P*=0.64).

### Detection of CTC in peripheral blood

**Figure 6:**
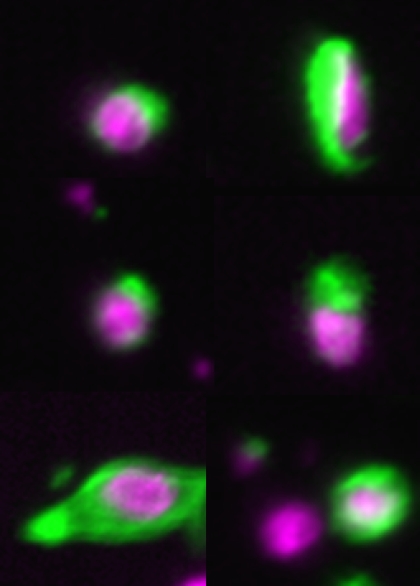
Images of circulating tumour cells from the CellTracks Analyzer II obtained from 7.5 ml of blood from breast cancer patients.

Overall, positive cells were identified in the blood of 65% of breast cancer patients and 10% of controls (Figure 6). Numbers of CTC were significantly higher in blood samples of patients with breast cancer than in healthy controls: the median number of CTC detected in 7.5 ml of blood was 1 (range, 0–2617) in breast cancer patients and 0 (range 0–1) in controls (*P*<0.001). Numbers of CTC also tended to differ between different patients groups, although this was not statistically significant: no CTC were detected in group A, the median number of CTC in group B was 1 (0–2617) and the median number of CTC in group C was 0.5 (0–153) (*P*=0.07). CTC numbers by the CellSearch System were significantly correlated with the patients' age (*r*=0.380, *P*=0.001) and CA15.3 levels (*r*=0.685, *P*<0.001). Furthermore, the number of CTC tended to be higher in ER+ breast tumours (*P*=0.09) and HER2– breast tumours (*P*=0.08), although this was not statistically significant.

Using ⩾2 cells as a threshold for positive samples in the patient population, which corresponded to 100% specificity in the control population, 34% of samples from patients were positive with the CellSearch System. A significant association was observed between CTC and tumour progression: in 22 of 46 (48%) patients with progressive disease CTC were detected, whereas only in 5 of 26 (9%) patients with stable disease CTC were present (*P*=0.02) (Table 3c).

### Association between CTC and total plasma DNA or serum methylated DNA

**Figure 7:**
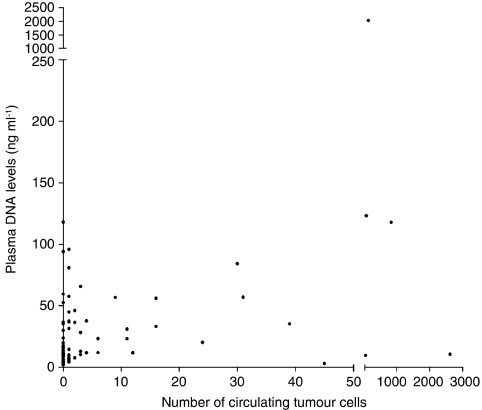
Correlation between plasma total DNA levels and the number of circulating tumour cells in the blood from breast cancer patients.

The concentration of total DNA in plasma was significantly correlated with the number of CTC detected in peripheral blood (*r*=0.368, *P*=0.001) (Figure 7). Patients with CTC in peripheral blood had significantly higher plasma total DNA levels than patients with no CTC: median DNA concentrations were, respectively, 10 (range, 2–118) ng ml^–1^ and 31 (3–2027) ng ml^–1^ (*P*=0.002) (Table 5a). PMR values for *APC* (*r*=0.314, *P*=0.005) and *RASSF1A* (*r*=0.492, *P*<0.001) were significantly correlated with the number of CTC detected in peripheral blood. No associations were found between PMR values for *ESR1* and CTC. In patients with at least one gene methylated in serum (*n*=42), CTC were detected in 19 (45%) cases. In patients without any methylation in serum DNA (*n*=36), no CTC were detected in 28 (78%) cases. Detection of a methylated gene in serum was significantly associated with the detection of CTC in peripheral blood (*P*=0.03) (Table 5b). Also the number of methylated genes in serum was significantly correlated with the presence of CTC in paired specimens (*P*=0.01).

### CTC, total and/or methylated DNA as predictive markers of tumour progression

Next, we evaluated the correlation of the three molecular markers (high plasma total DNA, presence of CTC and methylated markers in serum) with tumour progression. Overall, 18% of breast cancer patients were positive for all the three molecular markers (high plasma DNA, at least one methylated gene in serum and ⩾2 CTC), 56% of breast cancer patients were positive for at least two molecular markers, 82% of breast cancer patients were positive for at least one molecular marker and 18% of breast cancer patients were negative for all three markers. Of 19 healthy controls, 13 (68%) were negative for all three markers. The presence of multiple markers was not detected in the control population.

Of all patients with stable disease (*n*=25), two (8%) were positive for all three markers, nine (36%) were positive for ⩾2 markers, 19 (76%) were positive for at least one marker and six (24%) were completely negative. In contrast, of all patients with progressive disease (*n*=46), 16 (35%) were positive for all the three markers, 34 (74%) were positive for ⩾2 markers, 42 (91%) were positive for at least one marker and four (9%) were completely negative (*P*=0.01) (Table 3d).

## Discussion

Circulating DNA is present in plasma in healthy controls and is increased in cancer patients (Leon *et al*, 1977). These findings have attracted much attention to the potential use of elevated concentrations of circulating total DNA as a tumour marker. In this study, we measured the level of circulating DNA in the plasma of healthy controls and patients with localised or metastatic breast cancer using a real-time quantitative PCR method. Our results show a 3.25-fold difference in the median levels of circulating total DNA in plasma between breast cancer patients and healthy controls. The ROC curve result shows a good association between high DNA concentration and malignancy. The range of total DNA levels in the circulation of breast cancer patients varied widely, from levels like those in the controls (2–17 ng ml^–1^) to levels that exceeded values of 2000 ng ml^–1^ plasma. The highest values were measured in patients presenting in our clinic with untreated metastatic disease. Furthermore, total DNA levels tended to correlate with the tumour serum marker CA15.3, which is a high molecular glycoprotein (mucin) that can be detected in the peripheral blood of breast cancer patients. Although in patients with primary breast cancer the concentration of CA15.3 is usually within the normal range, increased levels of CA15.3 are often observed in patients with metastatic disease and correlate with an increased metastatic load (Antoine *et al*, 1994).

Circulating nucleic acids harbouring tumour-specific alterations have been identified in serum or plasma from breast cancer patients (Silva *et al*, 1999a). Methylated DNA markers are attractive tumour markers in blood for several reasons: (a) DNA in blood is stable, is easy to obtain and can be analysed by a high-throughput method such as qMSP; (b) methylated DNA markers are frequently found in a wide range of human cancers and not (or rarely) in healthy controls (Silva *et al*, 1999a, 1999b; Wong *et al*, 1999; Goessl *et al*, 2000; Sanchez-Cespedes *et al*, 2000; (c) in general, a high concordance between epigenetic alterations in primary tumour specimens and in blood has been reported (Usadel *et al*, 2002; Hoque *et al*, 2004; Topaloglu *et al*, 2004; Yang *et al*, 2004); (d) they are not limited to patients with metastatic cancer, but are also present in body fluid from patients with early or organ-confined tumours (Esteller *et al*, 1999; Sanchez-Cespedes *et al*, 2000). As no single gene is known to be hypermethylated in more than a proportion of breast tumours, it is necessary to use a panel of genes to provide a target for detection in serum. We selected three genes, namely, *APC*, *RASSF1A* and *ESR1*, which are known to be frequently hypermethylated in breast cancer (Widschwendter and Jones, 2002). We decided to measure methylation levels of these genes in serum instead of plasma, as Müller *et al* (2003) indicated the prognostic relevance of methylated DNA coding for these three genes in pre-therapeutic serum samples of patients with primary breast cancer (Müller *et al* 2003). Furthermore, to date, their remains no consensus as to whether plasma or serum is better for the assessment of circulating methylated DNA in cancer patients' blood. Overall, 53% of breast cancer patients showed hypermethylation of at least one of the three genes. *RASSF1A* had the highest frequency of hypermethylation with 35% of breast cancer cases being positive, followed by *APC* and *ESR1* being methylated in 29 and 20% of breast cancer cases, respectively. Methylation in serum DNA was never detected if this alteration was not present in the primary tumour tissue. We found no correlation between methylated DNA levels in serum and corresponding levels in the primary tumour tissue. This could be because of the specific physiological characteristics in the progression of each tumour, for example, angiogenic capacity, the ability to cause local necrosis (Usadel *et al*, 2002; Widschwendter *et al*, 2004a). Alternatively, the quantity and quality of DNA templates extracted from serum or the original primary tumour can differ on the basis of time of collection, the content of DNAse and other factors (Usadel *et al*, 2002; Widschwendter *et al*, 2004a) or the presence of normal DNA in blood may obscure minute amounts of circulating tumour-related DNA.

Methylation was also detected in a small proportion of controls (16%). Methylation of several genes has been reported earlier in non-malignant tissues and serum DNA of smokers. Detection of methylation in serum DNA could be a marker of disease (an early neoplastic effect), exposure (a biological effect of any environmental factor) or both (Hoque *et al*, 2006).

Our most interesting finding of correlating methylation data to clinicopathological variables was the strong association between methylation of *RASSF1A* in serum DNA and HR status. Methylation of *RASSF1A* in breast cancer has already been connected to hormone regulation, but the mechanism is not clear yet. Widschwendter *et al*. reported significant differences in the HR status between clusters of DNA methylation profiles (Widschwendter *et al*, 2004b). Feng *et al*. investigated whether methylation of a set of growth-suppressor genes would correlate with the expression of ER and PR, and found that methylation of *RASSF1A* was strongly correlated with ER, PR and HR expression (Feng *et al*, 2007). Also in the study by Sunami *et al*., hypermethylation of *RASSF1A* was more frequently present in ER+ breast tumours than in ER– breast tumours (Sunami *et al*, 2008). Thus, it seems that epigenetic alterations in the *RASSF1A* gene promoter and HR regulation in breast cancer are tightly linked.

Using the CellSearch System, at least one positive cell was identified in the blood of 65% of breast cancer patients and 10% of controls. It has been reported that false positive results using immuno-mediated CTC detection techniques can occur by specific labelling of non-tumour epithelial cells or non-specific labelling of non-tumour non-epithelial cells (Paterlini-Brechot and Benali, 2007). Variable numbers of epithelial cells have been found in peripheral blood of subjects without malignancy, being related to benign epithelial proliferative diseases, inflammation, tissue trauma, semi-surgical and surgical interventions (Crisan *et al*, 2000; Goeminne *et al*, 2000; Fehm *et al*, 2005).

To date, there is no agreement on the mechanisms that are responsible for the presence of tumour DNA shed into the bloodstream. The most common hypothesis is the shedding of lysed CTC. However, this seems to be unlikely because the number of CTC is inadequate to explain the observed amount of DNA in the plasma or serum (Stroun *et al*, 2000). Other possibilities are that apoptosis and necrosis of tumour cells increase the levels of circulating DNA (Jahr *et al*, 2001) or that DNA is actively released into the bloodstream by the tumour (Stroun *et al*, 2000). In this study, we observed a good association between total DNA or tumour-specific DNA levels and the number of CTC in blood from breast cancer patients. Patients with CTC in peripheral blood had significantly higher plasma total DNA levels than patients with no CTC. PMR values for *APC* and *RASSF1A* were significantly correlated with the number of CTC detected in peripheral blood. Furthermore, a combination assessment of these three molecular markers could predict tumour progression. Similar findings to ours have been reported earlier in patients with melanoma (Koyanagi *et al*, 2006). Koyanagi *et al*. showed that the detection of CTC was correlated with tumour-related methylated DNA and that a combined assessment of both molecular markers improved the assessment of prognosis in stage IV melanoma patients treated with biochemotherapy. However, Schwarzenbach *et al*. did not observe a correlation between the incidence of loss of heterozygosity in circulating DNA and the presence of CTC in the blood from breast cancer patients (Schwarzenbach *et al*, 2004). The observed correlation between CTC and circulating methylated DNA in our study could be interpreted in two ways: (a) CTC are a potential source of circulating tumour-specific DNA; (b) high numbers of CTC and circulating methylated DNA are both a phenotypic feature of more aggressive tumour biology.

In conclusion, this study provides evidence that the detection of large amounts of free circulating total DNA and of methylated genes are associated with CTC in peripheral blood from patients with advanced breast cancer. Furthermore, the combined assessment of all three molecular markers was predictive for tumour progression. However, large-scale studies are necessary to verify the clinical utility of CTC and serum methylated DNA as potential prognostic factors in breast cancer patients.

## Figures and Tables

**Table 1:** Frequency of methylated genes (% positives) according to clinicopathological features

**Variables**	**Number of patients**	***APC***	***RASSF1A***	***ESR1***	**At least one gene methylated**
*ER*					
Positive	53	36	47^*^	19	62^*^
Negative	22	18	14	23	36
					
*PR*					
Positive	38	32	42	16	58
Negative	35	31	34	26	54
					
*HR*					
Positive	52	37	48^*^	19	63^*^
Negative	21	19	14	24	38
					
*HER2*					
Positive	26	35	31	15	58
Negative	52	27	38	21	50
					
*Triple negative*					
Positive	10	20	20	20	30
Negative	63	33	41	21	60
					
*p53*					
Positive	18	39	33	22	55
Negative	33	33	45	24	70
					
*Disease status*					
PD	46	39	52^*^	26	67^*^
SD	26	19	12	15	38

Abbreviations: APC=adenomatous polyposis coli; ESR1=oestrogen receptor 1; HR=hormone receptor; PD=progressive disease; RASSF1A=ras association domain family protein 1A; SD=stable disease.

^*^P-values ⩽0.05.

**Table 2:** Sensitivity and specificity of plasma DNA quantification assay

**Cut-off points (ng ml^–1^)**	**Sensitivity (%)**	**Specificity (%)**
6.1300	82.5	75
7.4700	76.3	80
8.2750	72.5	85
9.7350	65	90
12.2650	52.5	95

**Table 3:** Correlations of (a) plasma total DNA, (b) serum methylated DNA, (c) CTC and (d) molecular markers

	**Response**		
**(a) Total DNA levels**	**Stable (*n*=27)**	**Progressive (*n*=46)**	***P*-value**
High	16	39	0.01
Low	11	7	
			
**(b) Methylated serum DNA**	**Stable (*n*=26)**	**Progressive (*n*=46)**	
Methylated	10	31	0.02
Unmethylated	16	15	
			
**(c) CTC**	**Stable (*n*=26)**	**Progressive (*n*=46)**	
Positive	5	22	0.02
Negative	21	24	
			
**(d) Molecular markers**	**Stable (*n*=25)**	**Progressive (*n*=46)**	
Negative	6	4	0.01
At least one marker positive	10	8	
Two or more markers positive	7	18	
Three markers positive	2	16	

CTC=circulating tumour cell.

**Table 4:** Aberrant methylation of genes by age

	**Age (years)**				
**Genes**	**<40 (*n*=4)**	**40–49 (*n*=14)**	**50–59 (*n*=17)**	**⩾60 (n=44)**	***P*-value**
*APC*	0	3 (13%)	4 (17%)	16 (70%)	0.33
*RASSF1A*	1 (4%)	5 (18%)	4 (14%)	18 (64%)	0.61
*ESR1*	0	5 (31%)	3 (19%)	8 (50%)	0.35
At least one gene methylated	1 (2%)	6 (14%)	8 (19%)	27 (64%)	0.35

Abbreviations: APC=adenomatous polyposis coli; ESR1=oestrogen receptor 1; RASSF1A=ras association domain family protein 1A.

**Table 5:** (a) Correlation of plasma total DNA levels with CTC; (b) correlation of serum methylated DNA with CTC

	**CTC**		
**(a) Total DNA levels (ngml^−1^)**	**Negative (*n*=52)**	**Positive (*n*=27)**	***P*-value**
Median	10	31	0.002
Range	2–118	3–2027	
			
	**CTC**		
**(b) Methylated serum DNA**	**Negative (*n*=51)**	**Positive (*n*=27)**	
*APC*			
Methylated	11 (48%)	12 (52%)	0.03
Unmethylated	40 (73%)	15 (27%)	
			
*RASSF1A*			
Methylated	10 (36%)	18 (64%)	<0.001
Unmethylated	41 (82%)	9 (18%)	
			
*ESR1*			
Methylated	10 (62%)	6 (38%)	0.79
Unmethylated	41 (66%)	21 (34%)	

Abbreviations: APC=adenomatous polyposis coli; CTC=circulating tumour cell; ESR1=oestrogen receptor 1; RASS1A=ras association domain family protein 1A.
